# TCM nonpharmacological interventions for ankylosing spondylitis: A protocol for systematic review and network meta-analysis

**DOI:** 10.1097/MD.0000000000024279

**Published:** 2021-02-12

**Authors:** Haiyan Wang, Haiyang Yu, Tao Wang, Naijia Liu, Xiaogang Zhang, Qinling Wei, Jiexiang Tian

**Affiliations:** a Department of Acupuncture and Moxibustion, Affiliated hospital of Gansu University of Traditional Chinese Medicine, Lanzhou, Gansu Province; b College of Acupuncture and Tuina, Chengdu University of Traditional Chinese Medicine, Chengdu, Sichuan Province; c Department of Orthopedics, Affiliated Hospital of Gansu University of Traditional Chinese Medicine; d Clinical College of Traditional Chinese Medicine, Gansu University of Traditional Chinese Medicine; e Department of Rheumatism, Affiliated Hospital of Gansu University of Traditional Chinese Medicine, Lanzhou, Gansu Province, China.

**Keywords:** ankylosing spondylitis, protocol, systematic review, TCM nonpharmacological interventions

## Abstract

**Background:**

Ankylosing spondylitis (AS) is a common infammatory rheumatic disease that affects the axial skeleton. Traditional Chinese medicine (TCM) nonpharmacological interventions are gaining an increasing popularity for AS. Nevertheless, the evidence of efficacy and safety of random controlled trials (RCTs) remains controversial. This study aims to evaluate the efficacy and acceptability of different TCM nonpharmacological therapies by systematic review and network meta-analysis.

**Methods:**

According to the strategy, the authors will retrieve a total of 7 electronic databases by December 2020, including PubMed, the Cochrane Library, EMbase, China National Knowledge Infrastructure, China Biological Medicine, Chongqing VIP, and Wan-fang databases After a series of screening, 2 researchers will use Aggregate Data Drug Information System and Stata software to analyze the data extracted from the randomized controlled trials of TCM nonpharmacological interventions for AS. The primary outcome will be the improvement of Pain intensity and functional status/disability and the secondary outcomes will include lobal improvement, health-related quality of life, satisfaction with treatment, and adverse events. Both classical meta-analysis and network meta-analysis will be implemented to investigate direct and indirect evidences on this topic. The quality of the evidence will be evaluated using the Grading of Recommendations Assessment, Development and Evaluation instrument.

**Results:**

This study will provide a reliable evidence for the selection of TCM nonpharmacological therapies in the treatment of AS.

**Conclusion:**

This study will generate evidence for different TCM nonpharmacological therapies for AS and provide a decision-making reference for clinical research.

**Ethics and dissemination:**

This study does not require ethical approval. The results will be disseminated through a peer-reviewed publication.

**OSF registration number:**

DOI 10.17605/OSF.IO/FHD2U

## 1 Introduction

Ankylosing spondylitis (AS) is a chronic inflammatory rheumatic disorder with joint damage primarily to the joints of the spine and the sacroiliac, leading to progressive bone fusion of the spine.^[1]^ An epidemiological survey has shown that the AS prevalence rates in the United States ranged from 0.2% to 0.55%, the estimated number of patients with AS was from 1.30 to 1.56 million in Europe and from 4.63 to 4.98 million in Asia,^[2,3]^ a Chinese study described that the average incidence is 0.2% to 0.3% among China.^[4]^ In addition, patients with AS had increased risks of multiple diseases such as chronic obstructive pulmonary disease, asthma, type 2 diabetes, stroke, cancer, and depression.^[5–10]^ AS carries an enormous economic burden from both direct (e.g., treatment) and indirect (e.g., lost work productivity) costs, an economic survey conducted in American has reported that the average direct medical expenses of AS were $2674 in the first year, while the indirect expenses were $4945.^[8]^

Currently, nonsteroidal anti-inflammatory drugs (NSAIDs), disease-modifying antirheumatic drugs (DMARDs), and tumor necrosis factor inhibitors (TNFi) remain the primary classes of medications for the treatment of AS.^[11]^ However, prolonged therapies with drugs such as NSAIDs may have potential cardiovascular, gastrointestinal, and renal risks.^[12,13]^ Furthermore, the cost of medicine has brought a large financial burden to AS patients and their families.^[14]^

Therefore, more and more AS patients have turned their attention to some other treatments, such as complementary and alternative medicine (CAM). Traditional Chinese medicine (TCM), as a main component of CAM based on current knowledge, has been widely applied in the management of chronic conditions including AS in the world.^[15,16]^

Acupuncture, as a traditional Chinese therapy, is a popular complementary and alternative therapy in the world.^[17,18]^ In recent years, several basic and clinical studies have provided evidence that acupuncture is beneficial for the treatment of AS.^[19–23]^ Moxibustion is a form of traditional Chinese medicine that has been widely used in East Asia for thousands of years.^[24]^ Moxibustion is one of the complementary and alternative therapies frequently used by patients with rheumatic diseases worldwide, especially in East Asian countries such as China. Moxibustion imparts both heat stimulation via infrared radiation and the pharmacological actions of its herbal components.^[25,26]^ Several systematic review and meta-analyses have shown that moxibustion is effective in relieving rheumatic diseases such as rheumatoid arthritis and AS.^[27,28]^ Various clinical trials and animal studies have been conducted to investigate the benefits and mechanisms of moxibustion for treating AS.^[29–32]^ Tai Chi(TC) combined both Chinese martial art and health regimens into a common set of core principles, movements, and exercises.^[33]^ Several previous studies have determined that tai chi is beneficial for balance control, flexibility, aerobic capacity, and improving immunity.^[34]^ Tai chi also has been determined to improve symptoms related to AS.^[35]^ A randomized clinical trial has shown the therapeutic effects of Tai Chi on AS.^[36]^

Despite its long history of use and clinical and experimental support, the effects of TCM nonpharmacological therapies for AS have not been fully validated. Additionally, systematic reviews and meta-analyses have not been done yet. Thus, this systematic review and meta-analysis aimed to assess the effects and safety of TCM nonpharmacological therapies on symptoms of AS.

## 2 Methods

### 2.1 Protocol and registration

This protocol follows the Preferred Reporting Items for Systematic Reviews and Meta-Analyses Protocols (PRISMA-P) guidelines.^[37]^ The network meta-analysis (NMA) protocol has been registered on Open Science Framework (OSF) platform (https://osf.io/kabvc/), registration number: DOI 10.17605/OSF.IO/FHD2U.

### 2.2 Eligibility criteria

#### 2.2.1 Type of participant

Participants with AS were eligible. All studies including patients with AS diagnosed by any set of criteria were eligible for inclusion, such as DSM-5, International Classification of Diseases (ICD-10), regardless of gender, educational background, nationality, or outpatient therapy or inpatient therapy.

#### 2.2.2 Type of interventions and comparators

Interventions in the treatment group will include any kinds of TCM nonpharmacological interventions for AS, including acupuncture, acupressure, cupping, moxibustion, tuina, tai chi, etc. We also include TCM nonpharmacological interventions in combination with other conservative treatments. However, combined interventions consisting of 3 or more therapies or with potential safety problems will be excluded. Control interventions will include no treatment, sham acupuncture and sham moxibustion, waiting list, oral drugs, any active treatment. Studies comparing the same kind of TCM nonpharmacological interventions, but with different sessions, acupoints selections will be taken as the identical node in network analysis.

#### 2.2.3 Type of outcomes

##### 2.2.3.1 Primary outcomes

The primary outcome of the study includes Bath Ankylosing Spondylitis Disease Activity Index,^[38]^ Bath Ankylosing Spondylitis Function Index,^[39]^ and Visual Analogue Scale.^[40]^

##### 2.2.3.2 Secondary outcomes

The secondary outcomes will include the following: AS Quality of Life index,^[41]^ Bath AS Patient Global Score,^[42]^ finger-to-floor distance, occiput to wall distance. Inflammatory markers: C-reactive protein, erythrocyte sedimentation rate. Side effects: satisfaction with treatment and adverse events.

#### 2.2.4 Study design

This study is a systematic review and network meta-analysis of RCTs with TCM nonpharmacological therapies on AS. This research will include all relevant RCTs using TCM nonpharmacological therapies for AS and the first period in randomized cross-over trials, regardless of publication status. Quasi-RCTs, review documents, clinical experience, and case reports were excluded. Moreover, we will only search English and Chinese literature in this study. And we will remove the studies without comparable baselines and duplicate publications.

### 2.3 Literature retrieval strategy

Computer retrieval of published RCTs of Traditional Chinese medicine nonpharmacological interventions for AS is conducted in PubMed, the Cochrane Library (issue 12, 2020), EMbase, China National Knowledge Infrastructure (CNKI), China Biological Medicine (CBM), Chongqing VIP, and Wan-fang databases. The time limit of document retrieval is from the establishment of each database to December 31, 2020. Use medical subject heading (MeSH) terms and key words to identify RCTs with the limitation of Chinese and English language. In addition, inclusive literature from the field and references from previous evaluations will be manually retrieved to find other potentially relevant articles. Chinese search terms mainly include: “ankylosing spondylitis”; English search words include “ankylosing spondylitis,” “AS,” “acupuncture,” “moxibustion,” “cupping,” “tui na,” “Tai Chi,” etc. Taking PubMed as an example, the initial retrieval strategy is shown in Table 1 and will be adjusted according to the specific database.

**Table 1:** Search strategy of the PubMed.

Number	Search terms
#1	Ankylosing spondylitis[Mesh]
#2	Ankylosing spondylitis[Title/Abstract] OR Back Pain, Back[Title/Abstract] OR Spine Ache[Title/Abstract] OR Aches, Low Back[Title/Abstract] OR Backache, Hip[Title/Abstract] OR Hip pain[Title/Abstract] OR osteoarthritis, Recurrent[Title/Abstract] OR Ankylosing spondylitis, Mechanical[Title/Abstract]
#3	#1 OR #2
#4	Spine[Title/Abstract]
#5	Medicine, Chinese Traditional [Mesh]
#6	Traditional Chinese Medicine [Title/Abstract] OR TCM[Title/Abstract]
#7	#5 OR #6
#8	Nonpharmacological interventions[Title/Abstract] OR Non-drug treatment[Title/Abstract] OR Acupuncture [Title/Abstract] OR Electro-acupuncture[Title/Abstract] OR Moxibustion acupuncture[Title/Abstract] OR Moxibustion[Title/Abstract] OR Acupoint[Title/Abstract] OR Taiqi therapy[Title/Abstract] OR Tuina[Title/Abstract] OR Qigong[Title/Abstract]
#9	randomized controlled trial[Publication Type]
#10	controlled clinical trial[Publication Type]
#11	randomized[Title/Abstract]
#12	randomly[Title/Abstract]
#13	#9 OR #10 OR #11 OR #12
#14	#3 AND #4 AND #7 AND #8 AND #13

### 2.4 Literature selection and data extraction

**Figure 1:**
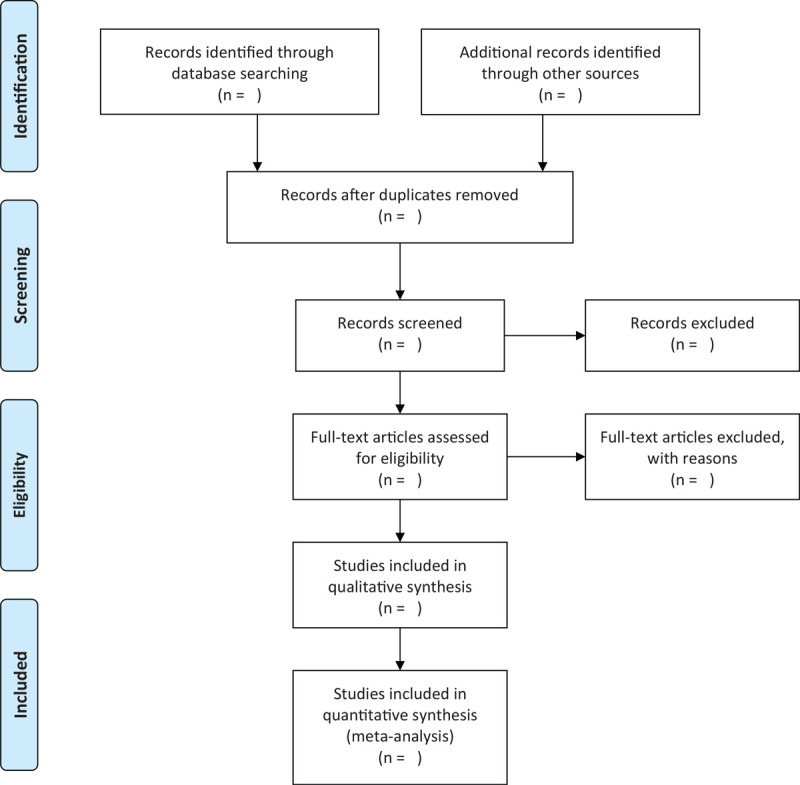
Flow chart of literature screening.

The study selection program will follow the Prisma guidelines, As shown in Figure 1, Haiyan Wang and Haiyang Yu will independently screen literatures according to inclusion and exclusion criteria and cross-checked against:

1. Preliminary screening of the literature through Endnote software to remove duplicates;
2. By reading the title and preliminarily screening the abstract, exclude the literature that obviously does not meet the inclusion criteria;
3. Download and read the full text for rescreening.

At the end of the filtering, the extracted features are recorded using a predesigned data table. These features include title, journal, author, publication year, country, sample size, gender, mean age, intervention, comparator, course of treatment, outcome measures, and follow-up time. If there is any disagreement, the third researcher Panju Cao will be asked to assist in the judgment. At the same time, the key factors of bias risk assessment are extracted.

### 2.5 Quality assessment

The quality of systematic review reflects the risk of bias or validity in its process and results, as well as the reliability of the included studies. The quality of the included studies will be assessed according to the Cochrane Reviewers’ Handbook. Two trained researchers Haiyang Yu and Tao Wang will independently evaluate the risk of bias of the included studies. If the results are disputed, they will be submitted to the corresponding author (Jiexiang Tian) of this study for review and determination.

Cochrane Reviewers’ Handbook will be used to assess the risk of RCTs being included in NMA, including^[43]^: random sequence generation; allocation concealment; blinding of the subjects and researchers; blinding of outcome assessment; incomplete outcome data; selective reporting; other bias.

### 2.6 Data synthesis and statistical methods

#### 2.6.1 Network meta-analysis

This study uses ADDIS 1.16.8 based on Bayesian framework for NMA.^[44]^ Odds ratios (ORs) or standardized mean differences will be modeled using Markov chain Monte Carlo methods, both with 95% confidence intervals (CIs). Preset model parameters: 4 chains are used for simulation analysis, with an initial value of 2.5, a step size of 10, 20,000 annealing times, and 50,000 simulation iterations. The network evidence plot will be generated according to different outcomes. According to the results of the NMA, rank probability plot of various TCM nonpharmacological therapies will be generated and sorted by dominance, with Rank 1 being the optimal sort.

#### 2.6.2 Consistency assessments/statistical model selection

The Node-split model is used to check for consistency between direct and indirect evidence. If there is no statistical difference (*P* > 0.05) between direct comparison and indirect comparison, the consistency model is used, whereas the inconsistency model is used for analysis. If the consistency model is adopted, then the stability of the results is verified by the inconsistency model: when the inconsistency factors including 0, at the same time inconsistency standard deviation including 1 says the result of consistency model is more stable and reliable. At the same time, various analysis models are iterated with preset parameters, and the convergence of iteration effect is judged by potential scale reduced factor (PSRF). When the PSRF value is close to or equal to 1 (1≤PSRF≤1.05), the convergence is complete, the model has good stability, and the conclusion of analysis is reliable. If the PSRF value is not in this range, the iteration continues manually until the PSRF value reaches the range standard.

#### 2.6.3 Heterogeneity test

Before the combination of effect size, we will use Stata to assess available study and patient characteristics to ensure similarity and to investigate the potential effect of heterogeneity on effect estimates. When inter-study heterogeneity exists, the random effect model is used. For comparison of each pair, heterogeneity is assessed by the statistic I^2^ value. When I^2^ > 50%, it indicates that there is heterogeneity between studies, and the source of heterogeneity should be further searched. When I^2^ < 50%, inter-study heterogeneity is considered to be small or there is no obvious heterogeneity.

#### 2.6.4 Sensitivity analysis

If necessary, the sensitivity analysis will be used to assess the effect of each study on the random effects model. The sensitivity of the general combined effect of all outcome indicators is analyzed by the exclusion method. That is, each study is excluded, and the remaining studies will be re-analyzed to identify the stability of the results. If there is no qualitative change in the combined effect showed in the results, the results are stable.

#### 2.6.5 Subgroup analysis

If necessary, we will conduct a subgroup analysis of duration of treatment, age, the course of AS, and research quality.

#### 2.6.6 Small sample effect/publication bias

If 10 or more studies are included in the NMA, a comparison-adjusted funnel plot is developed using Stata to evaluate the presence of small sample effects or publication bias in the intervention network. Descriptive analysis will be carried out through the symmetry of funnel plot. If the plot is asymmetric and there is no inverted funnel shape, it indicates that there may be publication bias. This may be related to the difficulty in the publication of the literature with negative results and the low quality of the inclusion methods.

#### 2.6.7 Dealing with missing data

If the required data is lost or incomplete, we will contact the corresponding author of the original document or the relevant email address of the first author. If there is no response, the record is excluded.

#### 2.6.8 Evaluating the quality of the evidence

To grade evidence quality and understand the current situation of evidence rating thereby analyzing possible problems, The Grading of Recommendations Assessment, Development and Evaluation (GRADE) instrument will be used to assess the quality of evidence in the NMA.^[45]^ Based on the risk of bias, inconsistency, imprecision, indirection, and publication bias, GRADE grades evidence quality into 4 levels: high, medium, low, and very low.

## 3 Discussions

Ankylosing spondylitis (AS) is the main form of chronic inflammatory arthritis affecting the axial skeleton and is characterized by excess spinal bone formation, inflammatory back pain, and radiographic sacroiliitis.^[1,2]^ AS is more common in young men with the characteristics of hidden onset, long course of the disease, and high disability rate, which has gradually become a serious public health problem.^[46]^ At present, adverse reactions and financial burdens are caused to patients by current medication treatment to a certain extent.^[16]^ In this situation, many people tend to use complementary and alternative therapies including TCM to treat AS. TCM therapy could have an indicative role in reducing the cases of AS. Therefore, we have to evaluate the efficacy and safety of TCM treatment.

With an increasing amount of publications on nonpharmacological interventions for patients with AS in recent years, we would like to figure out which has the relatively optimal effect and safety among those interventions. Given that systematic reviews with good quality can help provide best evidence in clinical practice, and a network meta-analysis can offer a ranking result based on comparative effectiveness, safety, and costs, we conceive and design this study protocol. We will assess the quality of evidence with the GRADE framework: risk of bias, heterogeneity or inconsistency, imprecision, indirectness, and publication bias. Our study will generate evidence of TCM in the treatment of AS and help to reduce the uncertainty about the effectiveness of AS management.

This study has a number of limitations. First, several studies we included were of low quality. Few RCTs comparing interventions and controls were available, limiting the number of studies that could be included in the meta-analysis. Second, since there were very few trails that had long-term follow-up, it is impossible to analyze the long-term effect. Last, the review may be susceptible to publication bias, though this was not evident when funnel plots were examined. As reported, data were markedly heterogeneous with a significant amount of unreported data.

## Author contributions

**Conceptualization:** Haiyan Wang, Haiyang Yu, Tao Wang

**Data curation:** Haiyan Wang, Haiyang Yu, Naija Liu.

**Formal analysis:** Xiaogang Zhang, Qinlin Wei.

**Funding acquisition:** Jiexiang Tian.

**Methodology:** Haiyan Wang, Haiyang Yu, Tao Wang.

**Project administration:** Haiyan Wang, Naija Liu.

**Writing – original draft**: Haiyan Wang, Haiyang Yu.

**Writing – review & editing:** Jiexiang Tian.

## Abbreviations

Abbreviations: ADDIS = Aggregate Data Drug Information System, AS = ankylosing spondylitis, GRADE = Grading of Recommendations Assessment, Development, and Evaluation, NMA = network meta-analysis, PSRF = potential scale-reduced factor, RCTs = randomized controlled trials, TCM = Traditional Chinese medicine.

## References

[1] Braun J Sieper J. Ankylosing spondylitis. Lancet 2007;369:1379–90. 17448825 10.1016/S0140-6736(07)60635-7

[2] Reveille JD Weisman MH. The epidemiology of back pain, axial spondyloarthritis and HLA-B27 in the United States. Am J Med Sci 2013;345:431–6. 23841117 10.1097/maj.0b013e318294457f PMC4122314

[3] Dean LE Jones GT MacDonald AG. Global prevalence of ankylosing spondylitis. Rheumatology (Oxford) 2014;53:650–7. 24324212 10.1093/rheumatology/ket387

[4] Zhao J Huang C Huang H. Prevalence of ankylosing spondylitis in a Chinese population: a systematic review and meta-analysis. Rheumatol Int 2020;40:859–72. 32125505 10.1007/s00296-020-04537-0

[5] Sharif K Watad A Tiosano S. The link between COPD and ankylosing spondylitis: a population based study. Eur J Intern Med 2018;53:62–5. 29631757 10.1016/j.ejim.2018.04.002

[6] Bremander A Petersson IF Bergman S. Population-based estimates of common comorbidities and cardiovascular disease in ankylosing spondylitis. Arthritis Care Res (Hoboken) 2011;63:550–6. 21452267 10.1002/acr.20408

[7] Meesters JJ Bremander A Bergman S. The risk for depression in patients with ankylosing spondylitis: a population-based cohort study. Arthritis Res Ther 2014;16:418. 25209603 10.1186/s13075-014-0418-z PMC4180137

[8] Sun LM Muo CH Liang JA. Increased risk of cancer for patients with ankylosing spondylitis: a nationwide population-based retrospective cohort study. Scand J Rheumatol 2014;43:301–6. 24559186 10.3109/03009742.2013.863969

[9] Chen HH Yeh SY Chen HY. Ankylosing spondylitis and other inflammatory spondyloarthritis increase the risk of developing type 2 diabetes in an Asian population. Rheumatol Int 2014;34:265–70. 24362789 10.1007/s00296-013-2927-5

[10] Keller JJ Hsu JL Lin SM. Increased risk of stroke among patients with ankylosing spondylitis: a population-based matched-cohort study. Rheumatol Int 2014;34:255–63. 24322454 10.1007/s00296-013-2912-z

[11] Ward MM Deodhar A Gensler LS. 2019 Update of the American College of Rheumatology/Spondylitis Association of America/Spondyloarthritis Research and Treatment Network Recommendations for the Treatment of Ankylosing Spondylitis and Nonradiographic Axial Spondyloarthritis. Arthritis Rheumatol 2019;71:1599–613. 31436036 10.1002/art.41042 PMC6764882

[12] Dougados M Béhier JM Jolchine I. Efficacy of celecoxib, a cyclooxygenase 2-specific inhibitor, in the treatment of ankylosing spondylitis: a six-week controlled study with comparison against placebo and against a conventional nonsteroidal antiinflammatory drug. Arthritis Rheum 2001;44:180–5. 11212158 10.1002/1529-0131(200101)44:1<180::AID-ANR24>3.0.CO;2-K

[13] Bonovas S Minozzi S Lytras T. Risk of malignancies using anti-TNF agents in rheumatoid arthritis, psoriatic arthritis, and ankylosing spondylitis: a systematic review and meta-analysis. Expert Opin Drug Saf 2016;15: (suppl): 35–54. 10.1080/14740338.2016.1238458 27924644

[14] Westhovens R Annemans L. Costs of drugs for treatment of rheumatic diseases. RMD Open 2016;2:e000259. 27651923 10.1136/rmdopen-2016-000259 PMC5020678

[15] Lv ZT Zhou X Chen AM. Akupunktur versus krankheitsmodifizierende Antirheumatika in der Behandlung der ankylosierenden Spondylitis--eine Metaanalyse [Acupuncture therapy versus disease-modifying antirheumatic drugs for the treatment of ankylosing spondylitis—a meta-analysis]. Forsch Komplementmed 2015;22:395–402. 26840422 10.1159/000442733

[16] Xuan Y Huang H Huang Y. The efficacy and safety of simple-needling therapy for treating ankylosing spondylitis: a systematic review and meta-analysis of randomized controlled trials. Evid Based Complement Alternat Med 2020;2020:4276380. 32617106 10.1155/2020/4276380 PMC7306850

[17] Kelly RB Willis J. Acupuncture for pain. Am Fam Physician 2019;100:89–96. 31305037

[18] Li C Pei Q Chen Y. The response-time relationship and covariate effects of acupuncture for chronic pain: a systematic review and model-based longitudinal meta-analysis. Eur J Pain 2020;24:1653–1665. 10.1002/ejp.1617 32533885

[19] Zhang D Liu W Yang H. [Clinical review of ankylosing spondylitis treated with acupuncture and medicine]. Zhongguo Zhen Jiu 2016;36:893–6. 29231581 10.13703/j.0255-2930.2016.08.032

[20] Chen Z Pang G. [Acupuncture assisted by dynamic moxibustion for adult ankylosing spondylitis at early-to-mid stage]. Zhongguo Zhen Jiu 2016;36:1157–60. 29231299 10.13703/j.0255-2930.2016.11.014

[21] Qin X Zhu B Zhang X. [Effects of”Tongdu Rezhen”method for ankylosing spondylitis at early stage: a randomized controlled trial]. Zhongguo Zhen Jiu 2016;36:793–6. 29231561 10.13703/j.0255-2930.2016.08.004

[22] Bai WJ Tan JL. [Observation on therapeutic effects of centro-square needling and triple needling on ankylosing spondylitis]. Zhongguo Zhen Jiu 2006;26:495–7. 16903602

[23] You Y Cai M Lin J. Efficacy of needle-knife combined with etanercept treatment regarding disease activity and hip joint function in ankylosing spondylitis patients with hip joint involvement: a randomized controlled study. Medicine (Baltimore) 2020;99:e20019. 32384461 10.1097/MD.0000000000020019 PMC7220523

[24] Shen X Ding G Wei J. An infrared radiation study of the biophysical characteristics of traditional moxibustion. Complement Ther Med 2006;14:213–9. 16911902 10.1016/j.ctim.2005.09.003

[25] Okada K Kawakita K. Analgesic action of acupuncture and moxibustion: a review of unique approaches in Japan. Evid Based Complement Alternat Med 2009;6:11–7. 10.1093/ecam/nem090 PMC2644273 18955231

[26] Kawakita K Shinbara H Imai K. How do acupuncture and moxibustion act? Focusing on the progress in Japanese acupuncture research. J Pharmacol Sci 2006;100:443–59. 16799260 10.1254/jphs.crj06004x

[27] Sun ZL Xu X Du SZ. Moxibustion for treating rheumatoid arthritis: a systematic review and metaanalysis of randomized controlled trials. Eur J Integr Med 2014;6:621–30.

[28] Hu J Mao Y Zhang Y. Moxibustion for the treatment of ankylosing spondylitis: a systematic review and meta-analysis. Ann Palliat Med 2020;9:709–20. 32312058 10.21037/apm.2020.02.31

[29] Zuo Z Liu Z Yuan K. [Effects and mechanism of the long-snake moxibustion on ankylosing spondylitis based on Th17/Treg/Th1 immune imbalance]. Zhongguo Zhen Jiu 2018;38:1053–7. 30672234 10.13703/j.0255-2930.2018.10.006

[30] Tian ZH Wang XY Zhang YF. Efficacy of herb-separated moxibustion combined with sulfasalazine enteric-coated tablets for ankylosing spondylitis with cold-dampness obstruction type. Zhongguo Zhen Jiu 2019;39:44–8. 30672255 10.13703/j.0255-2930.2019.01.010

[31] Hu H Li B Hu T. Analysis on the dominant diseases treated with spreading moxibustion therapy based on randomized controlled trials. Zhongguo Zhen Jiu 2019;39:557–61. 31099231 10.13703/j.0255-2930.2019.05.027

[32] Xu X Shi YN Wang RY. Metabolomic analysis of biochemical changes in the tissue and urine of proteoglycan-induced spondylitis in mice after treatment with moxibustion. Integr Med Res 2021;10:100428. 32953451 10.1016/j.imr.2020.100428 PMC7486606

[33] Klein PJ Adams WD. Comprehensive therapeutic benefits of Taiji: a critical review. Am J Phys Med Rehabil 2004;83:735–45. 15314540 10.1097/01.phm.0000137317.98890.74

[34] Irwin M Pike J Oxman M. Shingles immunity and health functioning in the elderly: Tai Chi Chih as a behavioral treatment. Evid Based Complement Alternat Med 2004;1:223–32. 15841255 10.1093/ecam/neh048 PMC538519

[35] Koh TC. Tai Chi and ankylosing spondylitis—a personal experience. Am J Chin Med 1982;10:59–61. 7183208 10.1142/S0192415X82000105

[36] Lee EN Kim YH Chung WT. Tai chi for disease activity and flexibility in patients with ankylosing spondylitis—a controlled clinical trial. Evid Based Complement Alternat Med 2008;5:457–62. 18955296 10.1093/ecam/nem048 PMC2586320

[37] Shamseer L Moher D Clarke M. Preferred reporting items for systematic review and meta-analysis protocols (PRISMA-P) 2015: elaboration and explanation [published correction appears in BMJ 2015;350:g7647. 10.1136/bmj.g7647 25555855

[38] Madsen OR. Stability of fatigue, pain, patient global assessment and the Bath Ankylosing Spondylitis Functional Index (BASFI) in spondyloarthropathy patients with stable disease according to the Bath Ankylosing Spondylitis Disease Activity Index (BASDAI). Rheumatol Int 2018;38:425–32. 29299630 10.1007/s00296-017-3920-1

[39] Calin A Garrett S Whitelock H. A new approach to defining functional ability in ankylosing spondylitis: the development of the Bath Ankylosing Spondylitis Functional Index. J Rheumatol 1994;21:2281–5. 7699629

[40] Breivik H. Fifty years on the Visual Analogue Scale (VAS) for pain-intensity is still good for acute pain. But multidimensional assessment is needed for chronic pain. Scand J Pain 2016;11:150–2. 28850458 10.1016/j.sjpain.2016.02.004

[41] Doward LC Spoorenberg A Cook SA. Development of the ASQoL: a quality of life instrument specific to ankylosing spondylitis. Ann Rheum Dis 2003;62:20–6. 12480664 10.1136/ard.62.1.20 PMC1754293

[42] Jones SD Steiner A Garrett SL. The Bath Ankylosing Spondylitis Patient Global Score (BAS-G). Br J Rheumatol 1996;35:66–71. 8624626 10.1093/rheumatology/35.1.66

[43] Higgins JPT Green S. Cochrane Handbook for Systematic Reviews of Interventions Version 5.1.0 [updated March 2011]. The Cochrane Collaboration 2011.

[44] Van Valkenhoef G Tervonen T Zwinkels T. ADDIS: a decision support system for evidence-based medicine. Decision Support Syst 2013;55:459–75.

[45] Guyatt G Oxman AD Akl EA. GRADE guidelines: 1. Introduction-GRADE evidence profiles and summary of findings tables. J Clin Epidemiol 2011;64:383–94. 21195583 10.1016/j.jclinepi.2010.04.026

[46] Fang F Jian P Lixiao X. Identification of potential transcriptomic markers in developing ankylosing spondylitis: a meta-analysis of gene expression profiles. Biomed Res Int 2015;2015:1–6. 10.1155/2015/826316 PMC4320922 25688367

